# Mother–Infant Face‐to‐Face Interactions Serve a Similar Function in Humans and Other Apes

**DOI:** 10.1111/desc.70019

**Published:** 2025-04-10

**Authors:** Federica Amici, Manuela Ersson‐Lembeck, Manfred Holodynski, Katja Liebal

**Affiliations:** ^1^ Faculty of Life Sciences, Institute of Biology Human Biology & Primate Cognition, Leipzig University Leipzig Germany; ^2^ Department of Comparative Cultural Psychology Max Planck Institute for Evolutionary Anthropology Leipzig Germany; ^3^ Department of Education and Psychology, Comparative Developmental Psychology Freie Universität Berlin Berlin Germany; ^4^ Faculty of Psychology/Sport and Exercise Studies, Institute for Psychology in Education University of Münster Münster Germany

**Keywords:** comparative developmental psychology, great apes, maternal behavior, *Pan*, small apes

## Abstract

In humans, mothers and infants often engage in face‐to‐face interactions, which are often considered crucial for the social transmission of information and the typical social and cognitive development of infants. In this study, we used a comparative developmental perspective to provide an assessment of mother–infant face‐to‐face interactions in several great and small ape species and to better understand which aspects of face‐to‐face interactions are shared by humans with other species. We conducted longitudinal behavioral observations on 48 mother–infant pairs from five different genera (i.e., *Hylobates*: *N* = 9; *Homo*: *N* = 10; *Nomascus*: *N* = 6; *Pan*: *N* = 18; *Symphalangus*: *N* = 5), when infants were 1, 6, and 12 months old. Generalized linear mixed models revealed differences across ape genera and through development in the probability that mothers and infants engaged in face‐to‐face interactions during the first year of the offsprings’ life. As predicted, these interactions were more likely when mothers and infants spent less time in physical contact, in communities usually characterized by more distal parenting styles (i.e., WEIRD humans), and when infants became older and thus motorically more independent. Overall, our findings suggest that face‐to‐face interactions were likely present in the common ancestor of humans and small apes, and likely serve a similar function across ape species.

## 1 Introduction

From the first months of development, human infants typically engage in face‐to‐face interactions with their mothers, exchanging looks and mutual gazes in different contexts (Csibra and Gergely 2009; de Klerk 2020; Farroni et al. 2002; Hamilton and Holler 2023; Ransom et al. 2022). However, these interactions are not uniquely human: In chimpanzees and macaques, mothers and infants also appear to engage in face‐to‐face interactions, mostly in nonagonistic contexts (Bard 1994; Bard et al. 2005; Dettmer, Kaburu, Byers et al. 2016; Dettmer, Kaburu, Simpson et al. 2016; Ferrari et al. 2009; Sclafani et al. 2023). Yet, comparative developmental studies on this topic are still scant, and it is not clear yet whether face‐to‐face interactions are widespread in species other than humans, whether they emerge early on during development, and which function they have. In this study, we will first discuss the importance of face‐to‐face interactions during human development. We will use a broad operational definition of face‐to‐face interactions, including all instances of mutual gazes (i.e., mother and infant align their faces and look at each other, implying social engagement), but also infants’ and mothers’ looks (i.e., mothers or infants direct their gaze toward the other's head, regardless of any other form of social engagement or communicative exchange). We will then examine cross‐cultural and comparative findings challenging the idea that face‐to‐face interactions universally define human development. We will further discuss the benefits of comparative developmental perspectives to trace back the evolutionary roots of parenting in humans. Finally, we will introduce the main aims and hypotheses of our study, which relied on a comparative developmental approach to assess the presence, emergence, and possible function of face‐to‐face interactions in 48 mother–infant pairs of captive apes, during the first year of development.

**Public Significance Statement**

This study provides the first direct comparison of face‐to‐face interactions in five ape genera, including humans. Face‐to‐face interactions, including mutual gaze, were common in all genera, especially when mothers and infants were not in physical contact, suggesting that they serve a similar function across ape species.

**Summary**

- Mothers and infants often engage in face‐to‐face interactions, not only in humans but also in other great and small ape species.
- In apes, including humans, face‐to‐face interactions are more likely when mothers and infants are not in physical contact.
- In apes, including humans, face‐to‐face interactions are more likely when infants become older and more independent.
- Face‐to‐face interactions were likely present in the common ancestor of humans and small apes, and likely serve a similar function across ape species.

In humans, face‐to‐face interactions play a crucial role: From social learning processes to conversation and play, they are usually thought to provide an ideal context for the social transmission of information (Csibra and Gergely 2009; de Klerk 2020; Hamilton and Holler 2023; Ransom et al. 2022). Humans appear to have an innate predisposition to engage in this form of interactions (e.g., Batki et al. 2000; Farroni et al. 2002; Murray et al. 2016). Newborns, for instance, prefer to look at faces that engage them in mutual gaze, as compared to faces with averted gaze (Farroni et al. 2002). Moreover, from an early age, infants are often involved by mothers in face‐to‐face interactions, which are thought to promote the establishment of joint attention through a variety of verbal and nonverbal behaviors (Butterworth and Jarrett 1991; Farroni et al. 2002). Indeed, early exposure to face‐to‐face interactions is often considered essential for the typical social and cognitive development of infants, as it fosters the emergence of linguistic skills and facilitates phenomena like affect regulation, affective and social attunement, perspective taking and coordination, among others (e.g., Feldman 2007; Kleinke 1986; Kochanska et al. 1999; Masek et al. 2021; Murray et al. 2016; Stern 1974; Stern et al. 1985; Tamis‐LeMonda and Bornstein 1989).

In the last decades, however, cross‐cultural and comparative research have challenged the idea that face‐to‐face interactions universally define human development. First, despite sharing the belief that infants need to be nurtured and protected (Bornstein 2002), cultural communities largely differ in how this belief is instantiated during mother–infant interactions (Keller et al. 2004, 2005, Keller et al. 2009). In WEIRD (i.e., western, educated, industrialized, rich and democratic; Henrich et al. 2010) communities, for example, mothers typically show more distal parenting styles, which are characterized by a high frequency of face‐to‐face interactions and little body contact with their infants; in non‐western communities, however, mothers show more proximal parenting styles, they often engage in body contact, and face‐to‐face interactions are less common (Kärtner et al. 2010, Kärtner et al. 2013; Keller et al. 2004, 2005, Keller et al. 2009; Keller and Chaudhary 2017). In these communities, mutual gazes happen less frequently and might be less relevant to provide comfort to mothers and infants during physical separations (Keller 2007; Morelli et al. 2017), and joint engagement may be achieved in other modalities, without the need to establish reciprocal visual contact (Bard et al. 2021). Across cultures, there are differences not only in the frequency of face‐to‐face interactions, but also in the way they are used. Face‐to‐face interactions generally create an environment that fosters social engagement and communicative exchange, but the extent to which they are used to regulate infants’ behavior (Bornstein et al. 2012; Liu et al. 2013) or attention (Cote et al. 2023) might vary across cultural contexts.

Second, face‐to‐face interactions are not uniquely human but are also found in closely related species, such as nonhuman primates (hereafter, primates; Emery 2000). In primates, eye contact is often an implicit signal of threat, but in some species, individuals show more tolerance to it (Harrod et al. 2020). Moreover, primate mothers may engage in face‐to‐face interactions and mutual gazes with their infants, typically in nonagonistic contexts like grooming or social play, suggesting a positive social function of these behaviors, like in humans (Bard 1994; Bard et al. 2005). In captive chimpanzees, for instance, mothers engage in face‐to‐face interactions with their infants from the second week of age (Bard 1994), with frequencies of mutual gaze varying between 8 and 30 events per hour around 3 months of age, depending on maternal experience (Bard 1994; Bard et al. 2005). Moreover, as in humans, chimpanzee mothers who spend more time in physical contact with their infants are less likely to engage in mutual gaze, suggesting that face‐to‐face interactions might have a similar function in both species, providing comfort during separation (Bard et al. 2005). Direct comparisons of captive chimpanzees and WEIRD humans further show that, while mutual gazes are overall longer and more frequent in human than chimpanzee mother–infant pairs, face‐to‐face interactions are common in both species during the first year of infants’ development (Amici et al. 2023).

Face‐to‐face interactions between mothers and infants are not limited to chimpanzees but are also present in some monkey species, including wild black‐striped capuchins (*Sapajus libidinosus*), where they often occur during physical contact, promoting affective communication between mothers and infants, or more seldomly during separation, fostering the resumption of physical contact (Verderane et al. 2020). In rhesus macaques (*Macaca mulatta*), mothers and infants often engage in socio‐emotional interactions while keeping their faces aligned and engaging in mutual gaze and lip‐smacking (Dettmer, Kaburu, Byers et al. 2016; Ferrari et al. 2009). Infant macaques that more frequently engage in face‐to‐face interactions with their mothers are also more likely to interact with peers during their first year of life, suggesting that these interactions might foster infants’ social competence (Dettmer, Kaburu, Simpson et al. 2016). Moreover, like in humans, macaque mothers use specific mirroring responses when interacting with their infants (Sclafani et al. 2023). In apes other than chimpanzees, literature is scanter. Liebal et al. (2024) provided a first preliminary assessment of face‐to‐face interactions when describing parenting behavior in mother–infant pairs, including 11 human pairs, 21 pairs of chimpanzees and bonobos, and 20 hylobatid pairs of seven different species. Given their different research focus, however, Liebal et al. (2024) did not provide a fine‐grained assessment of face‐to‐face interactions in apes, as these were coded whenever mothers and infants aligned their faces for at least 5 s within a 10‐s interval, following a well‐established protocol in human literature (e.g., Keller et al. 2009) that, however, does not allow capturing shorter interactions. This is especially problematic if one considers that apes often glance very shortly at each other (e.g., Kano et al. 2009; Kano et al. 2012) and that face‐to‐face interactions in apes might happen within shorter time frames, as compared to humans (see Bard et al. 2005).

In this study, we combined the comparative and developmental perspectives to assess mother–infant face‐to‐face interactions in several ape species that had seldomly been studied with regard to this behavior (Liebal et al. 2024). Building on previous work comparing 10 mother–infant pairs of WEIRD humans and 10 pairs of captive chimpanzees (Amici et al. 2023), we extended our dataset to include other species being phylogenetically close to humans and chimpanzees and that have been largely understudied. Evolutionary approaches to the study of human behavior consider interspecific comparisons between phylogenetically close species as a powerful tool to shed light on the evolutionary origins of specific human traits. In particular, comparative perspectives allow understanding which behavioral patterns humans share with other species and, by identifying the common ancestor of species sharing the same patterns, when they most likely emerged during evolution. Here, we observed a total of 48 pairs from 10 ape species and five genera (i.e., *Homo, Hylobates, Nomascus, Pan*, and *Symphalangus*). Pairs were observed longitudinally, when infants were 1, 6, and 12 months old (see Table 1), to assess possible differences in their developmental trajectories of face‐to‐face interactions. As there is little literature on this topic in most of these species (Liebal et al. 2024), we based our predictions on previous studies on WEIRD humans and chimpanzees (Amici et al. 2023; Bard 1994; Bard et al. 2005). In contrast to previous work on apes (Liebal et al. 2024), we coded mother–infant interactions frame by frame, to capture all instances of infants’ looks, mothers’ looks, and mutual gazes, regardless of their duration (see Methods). Moreover, we specifically assessed the distance at which these interactions took place, to explore their use also in situations of physical separation.

**TABLE 1:** For each study subject, genus, species, identity, sex/gender (i.e., F: female: M: male), study location (i.e., BE: Berlin Zoo, BU: Burgers’ Zoo, DR: Drusillas Zoo Park; EB: Eberswalde Zoo, HE: Hellabrunn Zoo, JA: Jaderpark, KR: Kristiansand Zoo, LE: Leipzig Zoo, MU: Mulhouse Zoo, MÜ: Münster Zoo, NO: Noah's Ark Zoo, OS: Osnabrück Zoo, PA: Parken Zoo, PL: Planckendael Zoo, RA: Randers Regnskov Zoo, TW: Twycross Zoo, UL: Ulm Zoo, WI: Wilhelma Zoo, ZÜ: Zürich Zoo), age at which they were observed, and total observational effort (in minutes).

Genus	Species	Subject	Sex	Zoo	1 month	6 months	12 months
*Homo*	*H. sapiens*	An	M	—	X	X	X
		Hd	F	—	X	X	X
		Hl	F	—	X	X	X
		Hn	M	—	X	X	X
		Jl	F	—	X	X	X
		Jr	M	—	X	X	X
		Ld	M	—	X	X	X
		Ls	M	—	X	X	X
		Mn	F	—	X	X	X
		Pp	M	—	X	X	X
Observational effort (individual mean ± SD)					552 (55 ± 18)	570 (57 ± 10)	606 (61 ± 1)
*Hylobates*	*H. lar*	Chili	F	PA	X		
		Kairi	F	UL		X	X
		Kedua	F	WI		X	X
		Luke	M	BE		X	X
		Sholo	M	DR		X	X
	*H. moloch*	Mia	F	HE	X	X	X
	*H. pileatus*	Gismo	M	RA		X	
		Jantan	M	ZÜ	X		
		Laju	M	ZÜ		X	X
Observational effort (individual mean ± SD)					359 (40 ± 56)	816 (91 ± 49)	716 (80 ± 56)
*Nomascus*	*N. gabriellae*	Baby	F	EB	X	X	X
		Bobo	M	BU			X
		Firmin	M	MU	X		
	*N. leucogenys*	Curly	F	OS	X	X	X
		Mohio	M	PA		X	
	*N. siki*	Fengshui	M	MU		X	
Observational effort (individual mean ± SD)					351 (59 ± 59)	477 (79 ± 56)	327 (55 ± 55)
*Pan*	*P. paniscus*	Fimi	F	LE	X		
		Hongo	M	PL			X
		Huenda	F	PL		X	X
		Kasai	M	LE	X	X	
		Kivu	M	BE	X	X	X
		Loto	M	LE	X	X	X
		Luiza	F	LE	X	X	X
		Yaro	M	LE	X	X	
	*P. troglodytes*	Azibo	M	LE	X	X	X
		Bangolo	M	LE	X	X	X
		Kara	F	LE	X	X	X
		Kofi	M	LE	X	X	X
		Lobe	M	LE	X	X	X
		Lome	M	LE	X	X	X
		Mora	F	LE	X	X	
		Nayla	F	MÜ	X	X	
		Tai	F	LE	X	X	X
		Yara	F	KR	X	X	X
Observational effort (individual mean ± SD)					1648 (92 ± 71)	1759 (98 ± 67)	1197 (67 ± 67)
*Symphalangus*	*S. syndactylus*	Denzel	M	TW	X		
		Jai	M	JA	X	X	X
		Sam	M	NO	X	X	
		Stig	M	TW		X	
		Tiga	M	BU	X	X	
Observational effort (individual mean ± SD)					480 (96 ± 48)	468 (94 ± 47)	81 (16 ± 32)

Face‐to‐face‐interactions are known to have an important affiliative and communicative function in both humans (e.g., Bornstein et al. 2012; Masek et al. 2021; Murray et al. 2016) and other primate species (Bard 1994; Bard et al. 2005; Dettmer, Kaburu, Byers et al. 2016; Dettmer, Kaburu, Simpson et al. 2016; Ferrari et al. 2009; Verderane et al. 2020). During physical separation, in particular, face‐to‐face interactions might play a crucial role in facilitating communicative exchanges between mothers and infants, regulating infants’ behavior and attention and/or providing comfort during separation, as suggested in humans (Bornstein et al. 2012; Cote et al. 2023; Keller 2007; Lavelli and Fogel 2002; Liu et al. 2013; Morelli et al. 2017). Therefore, we hypothesized that face‐to‐face interactions would be present in all study groups, but their frequency would increase (1) when mothers and infants spent less time in body contact, (2) in species that overall had more distal parenting styles, and (3) when infants got older and became motorically more independent. In particular, we predicted that the probability of mother–infant face‐to‐face interactions (i.e., maternal looks, infants’ looks, and mutual gaze events) would increase with decreasing time spent in body contact (Prediction 1). Moreover, as WEIRD humans have more distal parenting styles than other ape species (Kärtner et al. 2010; Keller and Kärtner 2013; Keller et al. 2009), we predicted that they would more likely engage in face‐to‐face interactions as compared to *Hylobates, Nomascus, Pan*, and *Symphalangus* (Prediction 2). Finally, we predicted that, in all genera, mother–infant pairs would more likely engage in face‐to‐face interactions when infants became older and more mobile (Prediction 3a), although the onset of these changes might happen later in humans than in other apes (Prediction 3b), as their motoric development is slower (see Bründl et al. 2021).

## 2 Methods

**FIGURE 1:**
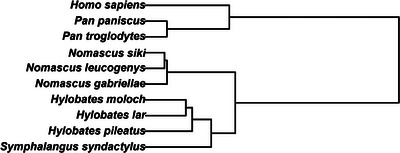
Phylogenetic tree of the study species, subsampled and pruned from vertlife.org (Upham et al. 2019). The length of the branches represents the estimated divergence time between species, which implies that species sharing shorter branches have a longer evolutionary history in common.

*Subjects*. We conducted behavioral observations on 48 mother–infant pairs from five different genera: *Hylobates* (*N* = 9), *Homo* (*N* = 10), *Nomascus* (*N* = 6), *Pan* (*N* = 18), and *Symphalangus* (*N* = 5) (Figure 1). Most pairs were observed longitudinally, when infants were 1, 6, and 12 months old, although some pairs could only be observed at one or two points in time. For more details about the species included and the characteristics of the pairs observed, see Table 1. Human pairs were recruited when mothers were in the third semester of their pregnancy or had infants younger than 1 month in two German cities (Berlin and Leipzig), through study advertisements and through the participant pool of the Excellence cluster “Languages of Emotion.” All mothers had a full‐time delivery and did not have postnatal depression. All the other apes lived in captivity in social groups and were housed in enclosures with indoor and outdoor areas with climbing structures and enrichment objects.

*Data collection*. We conducted behavioral observations via focal animal sampling (Altman 1974), video‐recording all focal samples with a video camera (Panasonic, HDC‐HS30), when infants were 1, 6, and/or 12 months of age. Although developmental patterns may clearly differ across species (see, e.g., Bründl et al. 2021), we focused on these ages because they correspond to crucial developmental milestones in humans. At 1 month of age, for instance, human infants start showing the first forms of social engagement, orienting their attention toward social and nonsocial stimuli and reliably seeking eye contact in social contexts (Salley et al. 2016; Zeifman et al. 1996); around 6 months of age, they show the first rudiments of joint attention (D'Entremont et al. 1997; Morales et al. 1998), start engaging in ostensive communication (Senju and Csibra 2008), and exploring their environment (Fogel 2011). At 12, they engage in triadic interactions requiring joint attention and show some understanding of others’ goals and intentions (Carpenter et al. 1998; Carpenter and Call 2013; Tomasello 2008; Fogel 2011; Liszkowski et al. 2008). Matching these developmental milestones with those of our ape study species was unfortunately not possible, as there are not enough studies on ape development (but see Bründl et al. 2021, on chimpanzees). Therefore, our comparison across species should be taken with caution, and more studies will be needed to define comparable developmental periods across ape species, as done for instance in macaques (see Sclafani et al. 2023).

We observed humans when infants were alone with their mothers (although fathers could occasionally be also present), and the other apes when they were in their social group, without entering their enclosure. For each subject and age point, we conducted several focal samples, which varied between 5 and 60 minutes. Given the logistic challenge of longitudinally observing many mother–infant pairs across several countries, the observational effort was not the same across individuals, age points, and genera (Table 1), although this was controlled for by including it as an offset term in the statistical models (see below).

*Data coding and analyses*. We used the free software Boris (Friard and Gamba 2016) to code all the videos. In particular, we coded all instances in which (i) mothers looked at their infants (i.e., directing their gaze toward the infant's head), (ii) infants looked at their mothers (i.e., directing their gaze toward the mother's head), and (iii) mothers and infants engaged in mutual gaze (i.e., simultaneously looking at each other, with their faces aligned). Coding was done frame by frame, without any temporal threshold to define looks or gazes: These were coded, regardless of their duration, whenever the mother and/or the infant directed their gaze toward the other, by either moving the head/eyes toward the other's head, pausing, and then continuing the movement in the same direction, or by moving the head/eyes toward the other's head and then toward a different direction, with or without a pause (e.g., the head/eyes moved back and forth). We coded all mothers’ and infants’ looks and all mutual gazes, regardless of whether they happened together to other communicative exchanges or otherwise implied social engagement, to gain a broader understanding of face‐to‐face interactions during ape development. Moreover, we coded the duration of time in which mothers and infants were in body contact (i.e., one mother's body part was in contact with any body part of the infant). Finally, we coded the duration of time during which pairs were visible (i.e., a measure of the observational effort; see Table 1), and the duration of time in which the direction of their looks was visible (and face‐to‐face interactions could thus be coded, as an observational effort to be included in the models).

To ensure interobserver reliability, an observer naïve to the aims of the study recoded 10% of all the videos (i.e., 18 out of 173 total hours of videos). Interobserver reliability was good for all the variables coded (i.e., Spearman's correlation for the duration of body contact: *ρ* = 0.95; for the duration of maternal looks: *ρ* = 0.80; for the duration of infants’ looks: *ρ* = 0.81; for the duration of mutual gaze: *ρ* = 0.85; all *N* = 35, *p *< 0.001). We prepared our dataset entering one line for the focal sample (*N* = 375), specifying the identity, genus, sex, and age of the infant observed, whether mothers looked at their infants, infants looked at their mothers, mothers and infants engaged in mutual gaze, and the time in which the direction of their looks was visible.

We used this dataset to run three generalized linear mixed models (Baayen et al. 2008) in R (R Core Team 2021; version 4.2.2), using the glmmTMB package (Magnusson et al. 2021). Our responses were the probability of maternal looks (Model 1), of infants’ looks (Model 2) and of mutual gaze events (Model 3). All models followed a binomial distribution and included the two‐way interaction of genus and infant's age (and their main terms). They also included the proportion of time spent in body contact as a test predictor, the infant's sex as control (because mothers might interact differently with their male and female offspring), and the infant's identity as random factor. We also included observational effort as offset term in the models, to control for the different time pairs were observed in each focal sample.

Each full model was then compared with likelihood ratio tests to a null model that included offset term, controls, and random factors, but no test predictors (Dobson and Barnett 2008). In case of a significant comparison, we assessed the significance of the interaction with the drop1 function, and in case of a nonsignificant result, we rerun the full model only including the main terms. We also removed the interaction from Model 2 and rerun the model only including genus and age as main terms, because the full model including the interaction failed to properly converge, likely due to quasi‐complete separation in the dataset (see Table 2). We finally used the emmeans package with Tukey adjustments (Lenth 2020) to compare the levels of the significant categorical variables. We checked the model assumptions using the DHARMa (Hartig 2022) and performance packages (Lüdecke et al. 2021), which revealed no convergence, overdispersion, or multicollinearity issues (maximum variance inflation factors across models = 1.17; Miles 2005) in any of the models presented.

**TABLE 2:** Mean (±SD) probability of maternal looks, infants’ looks, and mutual gazes during the focal observation, for each genus and age point.

Behavior	Genus	1 month	6 months	12 months
Probability of mothers looking towards infants	*Homo*	1.00 ± 0.00	1.00 ± 0.00	0.95 ± 0.21
	*Hylobates*	0.73 ± 0.44	0.79 ± 0.41	0.74 ± 0.44
	*Nomascus*	0.40 ± 0.49	0.60 ± 0.49	0.45 ± 0.50
	*Pan*	0.81 ± 0.39	0.87 ± 0.34	0.82 ± 0.39
	*Symphalangus*	0.68 ± 0.46	0.63 ± 0.48	0.25 ± 0.43
Probability of infants looking towards mothers	*Homo*	1.00 ± 0.00	1.00 ± 0.00	0.91 ± 0.29
	*Hylobates*	0.27 ± 0.44	0.71 ± 0.45	0.52 ± 0.50
	*Nomascus*	0.30 ± 0.46	0.65 ± 0.48	0.36 ± 0.48
	*Pan*	0.40 ± 0.49	0.62 ± 0.49	0.67 ± 0.47
	*Symphalangus*	0.42 ± 0.49	0.67 ± 0.47	0.00 ± 0.00
Probability of mutual gaze	*Homo*	1.00 ± 0.00	1.00 ± 0.00	0.91 ± 0.29
	*Hylobates*	0.13 ± 0.34	0.43 ± 0.49	0.35 ± 0.48
	*Nomascus*	0.10 ± 0.30	0.45 ± 0.50	0.27 ± 0.45
	*Pan*	0.35 ± 0.48	0.55 ± 0.50	0.64 ± 0.48
	*Symphalangus*	0.37 ± 0.48	0.42 ± 0.49	0.00 ± 0.00

## 3 Results

**FIGURE 2:**
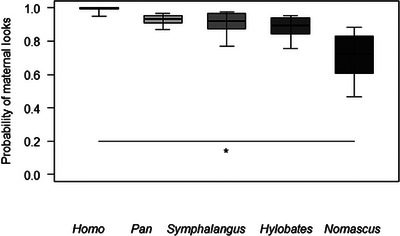
For each genus, the probability that mothers looked towards their infants during the focal sample. The thick horizontal lines represent the estimates for Model 1, which were averaged over the level of sex and age, and back‐transformed from the logit scale. The horizontal ends of the boxes represent the estimated marginal means ± SD, and the ends of the whiskers represent the 95% confidence intervals. Post hoc significant differences are marked with an asterisk.

In Table 2, we report the mean probability of maternal looks, infants’ looks, and mutual gaze events, for each genus and age point. For Model 1, the full model significantly differed from the null one, with a significant effect of the genus as the main term on the probability of maternal looks (Table 3). Post hoc tests showed that the probability of mothers looking toward infants was overall higher in *Homo* than *Nomascus* (*p* = 0.014; Figure 2).

**TABLE 3:** Results of the models run, including estimates, standard errors (SE), confidence intervals (CIs), likelihood ratio tests (LRT), degrees of freedom (*df*), and *p* values for all test predictors and controls (in italics); reference categories are in parentheses, and significant test predictors are marked with an asterisk (i.e., p≤0.05).

MODELS	Estimate	SE	2.5%–97.5% CI	LRT	*df*	*p*
Model 1: Probability of mothers looking towards infants (GLMM, χ^2^ = 18.34, *df* = 7, *p =* 0.011)						
Intercept	−7.30	1.41	−10.07 to −4.53	—	—	—
Genus (*Hylobates*)	−3.55	1.44	−6.37 to −0.73	17.65	4	0.001*
Genus (*Nomascus*)	−4.70	1.48	−7.59 to −1.80			
Genus (*Pan*)	−3.02	1.41	−5.80 to −0.25			
Genus (*Symphalangus*)	−3.20	1.52	−6.19 to −0.22			
Age (6 months)	0.39	0.42	−0.44 to 1.21	0.96	2	0.618
Age (12 months)	0.09	0.49	−0.87 to 1.05			
Proportion of time in body contact	0.03	0.43	−0.81 to 0.86	0.00	1	0.949
Sex (male)	−0.12	0.50	−1.11 to 0.87	0.06	1	0.813
Model 2: Probability of infants looking towards mothers (GLMM, χ^2^ = 57.30, *df* = 7, *p *< 0.001)						
Intercept	−8.14	1.01	−10.12 to −6.15	—	—	—
Genus (*Hylobates*)	−3.31	0.94	−5.15 to −1.47	24.53	4	<0.001*
Genus (*Nomascus*)	−3.29	0.98	−5.22 to −1.37			
Genus (*Pan*)	−3.10	0.92	−4.91 to −1.29			
Genus (*Symphalangus*)	−2.17	1.01	−4.15 to −0.18			
Age (6 months)	1.03	0.34	0.37 to 1.69	10.12	2	0.006*
Age (12 months)	0.50	0.44	−0.36 to 1.36			
Proportion of time in body contact	−1.66	0.64	−2.92 to −0.41	8.78	1	0.003*
Sex (male)	0.02	0.34	−0.65 to 0.69	0.00	1	0.950
Model 3: Probability of mothers and infants engaging in mutual gaze (GLMM, χ^2^ = 74.41, *df* = 15, *p *< 0.001)						
Intercept	−8.31	1.01	−10.30 to −6.32	—	—	—
Genus (*Hylobates*)	−4.45	0.97	−6.36 to −2.54	38.64	4	<0.001*
Genus (*Nomascus*)	−4.23	1.01	−6.21 to −2.25			
Genus (*Pan*)	−3.45	0.95	−5.31 to −1.60			
Genus (*Symphalangus*)	−3.12	1.04	−5.16 to −1.08			
Age (6 months)	0.80	0.34	0.13 to 1.46	5.68	2	0.058
Age (12 months)	0.73	0.44	−0.14 to 1.59			
Proportion of time in body contact	−1.45	0.64	−2.71 to −0.19	6.53		0.011*
Sex (male)	0.18	0.35	−0.51 to 0.88	0.28	1	0.598

**FIGURE 3:**
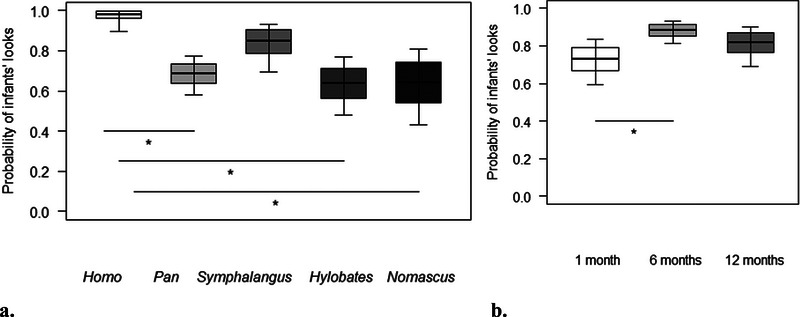
For each genus (a) and age (b), the probability that infants looked towards their mothers during the focal sample. The thick horizontal lines represent the estimates for Model 2, which were averaged over the level of sex and age (in a) and sex and genus (in b), and back‐transformed from the logit scale. The horizontal ends of the boxes represent the estimated marginal means ± SD, and the ends of the whiskers represent the 95% confidence intervals. Post hoc significant differences are marked with an asterisk.

For Model 2, we found a significant difference between the full and null models, with a significant effect of the proportion of time spent in body contact, and of genus and age as main terms on the probability of infants’ looks (Table 3). In particular, the probability of infants’ looks increased with decreasing time spent in body contact (Table 3). Post hoc tests further revealed that the probability of infants looking toward mothers was overall higher in *Homo* than in *Pan, Nomascus* (both *p* = 0.008), and *Hylobates* (*p =* 0.004; Figure 3a). Moreover, it increased for all genera from 1 to 6 months of age (*p < *0.001; Figure 3b).

**FIGURE 4:**
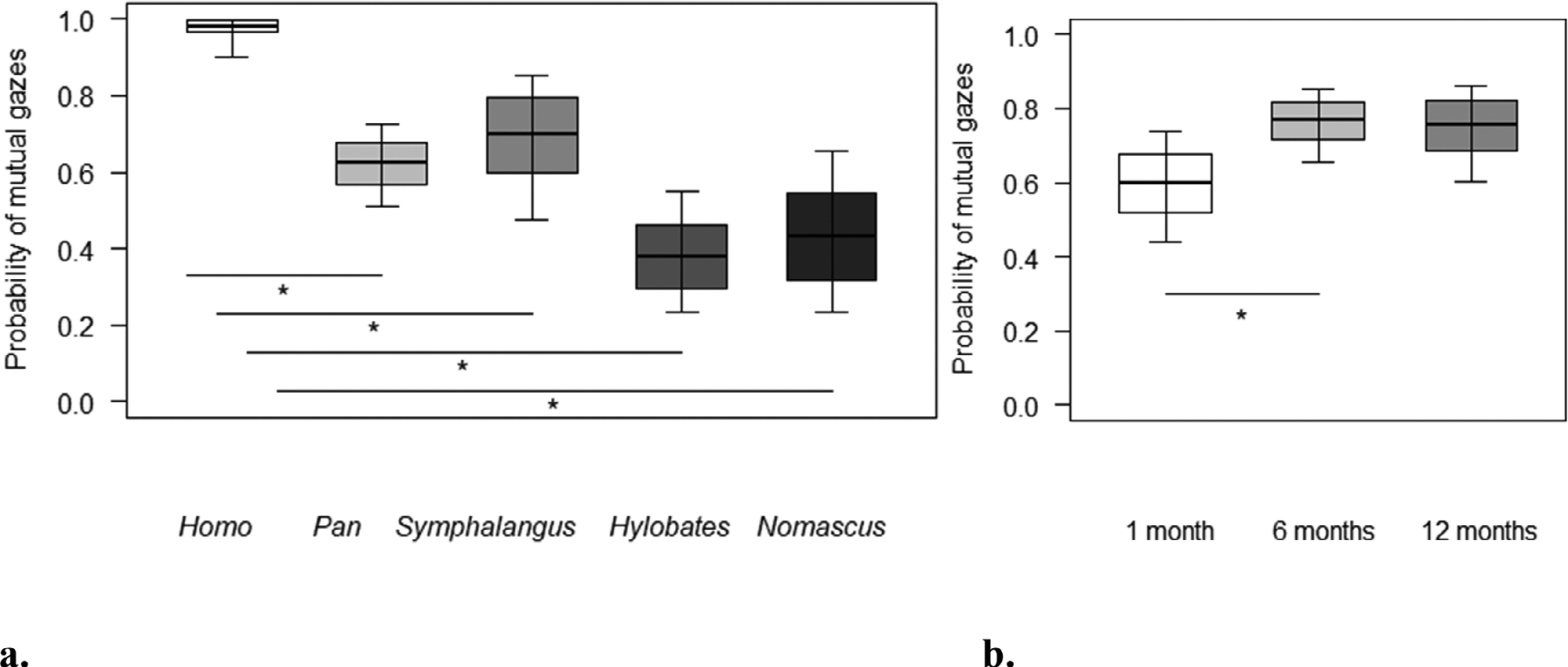
For each genus (a) and age (b), the probability that mothers and infants engaged in mutual gazes during the focal sample. The thick horizontal lines represent the estimates for Model 3, which were averaged over the level of sex and age (in a) and sex and genus (in b), and back‐transformed from the logit scale. The horizontal ends of the boxes represent the estimated marginal means ± SD, and the ends of the whiskers represent the 95% confidence intervals. Post hoc significant differences are marked with an asterisk.

Finally, for Model 3, we found a significant difference between the full and null models, with a significant effect of genus and proportion of time spent in body contact, and a marginal effect of age (Table 3), on the probability of mutual gaze events. In particular, the probability that mothers and infants engaged in mutual gaze increased with decreasing time spent in body contact (Table 3). Post hoc tests revealed that the probability of mutual gaze events was significantly higher in *Homo* than in all other genera (*Symphalangus*: *p =* 0.024; *Pan: p =* 0.003; *Hylobates* and *Nomascus*: *p* *<* 0.001; Figure 4a). Moreover, it marginally increased for all species from 1 to 6 months of age (*p =* 0.052; Figure 4b).

## 4 Discussion

Overall, our study found differences across ape genera and through development in the probability that mothers and infants engaged in face‐to‐face interactions during the first year of offspring's life. Largely in line with our predictions, these interactions were more likely when mothers and infants spent less time in physical contact, in study communities usually characterized by more distal parenting styles (i.e., WEIRD humans), and when infants became older and thus motorically more independent.

As predicted, we found an increase in the probability that infants would look toward their mothers and that they would engage in mutual gaze events, when the proportion of time spent in body contact with their mothers decreased (Prediction 1). This confirms and extends previous findings in humans (Keller 2007; Lavelli and Fogel 2002; Morelli et al. 2017) and chimpanzees (Bard et al. 2005), which showed a clear increase in face‐to‐face interactions with increasing physical separation. Therefore, although face‐to‐face interactions are more likely in some specific genera (see below), they appear to be used in a similar way by humans and other ape species, increasing with increasing distance (see Keller 2007; Morelli et al. 2017; Bard et al. 2005). In our study, physical separation increased the probability of infants looking at their mothers and engaging in mutual, but not of maternal looks, suggesting that mutual gazes during separation are mainly driven by infants looking at their mothers when not in physical contact.

Also in line with our predictions, our results showed that WEIRD humans, who are usually characterized by more distal maternal styles (Keller et al. 2004, 2005, Keller et al. 2009; Keller and Chaudhary 2017; Kärtner et al. 2013), were more likely than other genera to engage in face‐to‐face interactions (Prediction 2). In WEIRD humans, mothers were more likely to look toward their infants than in *Nomascus*, infants were more likely to look toward their mothers than in *Pan, Hylobates*, and *Nomascus*, and mutual gazes were more likely than in all the other genera. These results confirm and extend previous studies comparing WEIRD humans and chimpanzees, in which the former showed a higher frequency of mutual gaze events (Amici et al. 2023). Moreover, they are in line with other work suggesting that maternal looks are more frequent in humans than in other primate species (see Bard et al. 2005). Overall, there are two main conclusions that we can draw from these findings. First, physical separation appears to predict face‐to‐face interactions not only within individuals and species (depending on how close individuals are in a specific moment), but also more broadly, across genera, depending on their parenting styles. Second, the probability of mutual gaze events was higher in *Homo* than in all other genera, although differences in maternal and infants’ looks were not as strong (e.g., there were no differences in the probability of maternal and infants’ looks between *Homo* and *Symphalangus*). This suggests that the higher probability of mutual gazes in *Homo* did not simply depend on WEIRD mothers and infants more likely looking at each other, but also on better coordination of their gazing behavior (see Amici et al. 2023).

In line with Prediction 3a, we found that, across genera, the probability of face‐to‐face interactions overall increased as infants became older and consequently more mobile. However, in contrast to our Prediction 3b, there were no differences across genera with regards to the time at which this increase started, although motoric development is clearly not the same across ape species (e.g., Bründl et al. 2021). In particular, the probability of infants’ looks and marginally of mutual gaze events increased across genera from 1 to 6 months of infants’ age, when infants started spending more time away from their mothers. These changes over the first year of infants’ life remind us of the importance of including developmental perspectives in comparative studies, to detect differences and similarities across species in their developmental patterns (see Liebal and Haun 2018).

Overall, our study provides strong support to the hypothesis that mother–infant face‐to‐face interactions play a crucial role during physical separation, as they become more likely when mothers and infants spend less time in body contact, in species with more distal parenting styles, and when infants become more independent. Interestingly, this pattern is shared across ape genera, including humans, confirming that great and small apes share important aspects of their development with humans (see, e.g., Bard 1994; Rosati et al. 2014), although it is likely that, in humans, face‐to‐face interactions have a further unique function for the development of joint attention and social referencing. In the future, the direct comparison of apes with other human cultural communities, including non‐WEIRD ones, might be interesting to disentangle how much of the differences we currently evidenced between humans and other ape genera depend on differences that emerged during evolution or rather reflect cultural peculiarities of WEIRD communities. Indirect comparison with previous studies across human cultures, however, suggests that most of these differences are likely cultural (e.g., Keller et al. 2004, 2005, Keller et al. 2009; Keller and Chaudhary 2017; Kärtner et al. 2013).

As in humans, face‐to‐face interactions in primates are typically embedded in positive social contexts (Bard 1994; Bard et al. 2005; Dettmer, Kaburu, Byers et al. 2016; Dettmer, Kaburu, Simpson et al. 2016; Ferrari et al. 2009; Verderane et al. 2020). Yet, inferring their exact function during physical separation is no easy endeavor, and this study can only suggest some possible lines for future research. Mutual gaze and looks, for instance, may offer the ideal environment for communicative bouts and other forms of social engagement to take place, especially if the tactile modality is not available (Bard et al. 2005). Moreover, as in humans, face‐to‐face interactions might help mothers regulate infants’ behavior and attention (Bornstein et al. 2012; Cote et al. 2023; Liu et al. 2013) or provide comfort to both mothers and infants during separation (Keller 2007; Lavelli and Fogel 2002; Liu et al. 2013; Morelli et al. 2017). To really disentangle the function of face‐to‐face interactions during physical separation, future studies will have to assess which other forms of social engagement take place during face‐to‐face interactions, quantify the contextual occurrence of gestures, vocalizations and facial expressions, and assess whether face‐to‐face behaviors increase in response to the other's distress and result in its reduction.

Studies on mother–infant face‐to‐face interactions are extremely scant beyond humans, and often limited to few individuals (Amici et al. 2023) and short periods of time (Bard 1994; Bard et al. 2005). Therefore, by longitudinally following 48 mother–infant pairs from 10 primate species, including small apes, our study provides an important advance in the understanding of mother–infant face‐to‐face interactions. Nonetheless, our findings should be taken with caution, as our study also had several crucial limitations. First, despite including 48 mother–infant pairs, our dataset was still limited, constraining the level of detail that we could reach in our analyses. Our comparisons, for instance, were conducted across genera, rather than species, because finer‐grained analyses led to convergence issues in the models, due to the low number of observations for some species (see Table 1). Similarly, we limited our analyses to the probability of face‐to‐face interactions, although larger datasets might allow exploring other important aspects of these interactions, like their frequency and duration (see, e.g., Amici et al. 2023). In the same line, our relatively small sample size did not allow us to include the interaction between genus and age in Model 2 so that we cannot exclude different developmental patterns across genera in the probability of infants’ looks, although this is unlikely given the results of the other models. Second, the inclusion of relatively few pairs did not allow us to explore interindividual variation in face‐to‐face interactions, although these are likely important, given that maternal styles also strongly vary within primate species (e.g., Fairbanks 1996). In the same line, factors like offspring's sex or maternal experience might modulate some of the differences we have found (see, e.g., Bercovitch. et al. 1998; Bercovitch 2002; Kulik et al. 2016), but more data are needed to explore these complex interactions. Finally, our study only included captive primates, although apes in the wild might show different behavioral patterns. Face‐to‐face interactions, for example, might be more likely in captive apes than in wild ones, as the former experience more extensive contact with humans (Bard et al. 2005; Hopkins et al. 2020), so that special caution is needed when generalizing our findings to apes from other settings.

Overall, our study provides a preliminary comparison of mother–infant face‐to‐face interactions in WEIRD humans, great apes and small apes. By combining comparative and developmental perspectives, we showed that, despite variation across genera and through development, face‐to‐face interactions were common in all the genera observed, and generally decreased when mothers and infants spent less time in close proximity. Our findings suggest that face‐to‐face interactions were likely common already in the common ancestor of humans and small apes. Moreover, they suggest that face‐to‐face interactions likely play a similar function in apes during physical separation, and imply that differences between humans and other apes might be less clear‐cut than traditionally thought.

## Conflicts of Interest

The authors declare no conflicts of interest.

## Data Availability

The data that support the findings of this study are available from the corresponding author upon reasonable request.
